# Potential mechanism of Ziyin Tongluo Formula in the treatment of postmenopausal osteoporosis: based on network pharmacology and ovariectomized rat model

**DOI:** 10.1186/s13020-021-00503-5

**Published:** 2021-09-16

**Authors:** Rong-Bin Chen, Ying-Dong Yang, Kai Sun, Shan Liu, Wei Guo, Jin-Xin Zhang, Yong Li

**Affiliations:** 1 The Second Clinical Medicine College, Guangzhou University of Chinese Medicine, Guangzhou, 510006 China; 2 Guangdong Province Hospital of Traditional Chinese Medicine ZHUHAI BRANCH, 519015 Zhuhai, China; 3 Guangzhou University of Chinese Medicine, 510006 Guangzhou, China

**Keywords:** Postmenopausal osteoporosis, Ziyin Tongluo Formula, Traditional Chinese medicine, Ovariectomy, Network pharmacology, Molecular docking

## Abstract

**Background:**

Amending from ancient classic, Ziyin Tongluo Formula (ZYTLF) has been prescribed to treat postmenopausal osteoporosis (PMOP) for decades with good curative effect. However, the possible mechanisms of it are still unknown.

**Methods:**

Ovariectomized rat model was established to validate the therapeutic effect of ZYTLF on PMOP by Micro-CT bone analysis and pathological observation. Subsequently, active ingredients of ZYTLF and corresponding putative targets were identified by online databases. Overlapping genes were first obtained from mining genes associated with PMOP and then overlapped them with the putative targets. Key genes were selected from the multiple constructed and analyzed networks. GO and KEGG pathway enrichment analysis were performed by importing the key genes to the DAVID database. Moreover, validation of the binding association between key targets and their corresponding active compounds were accomplished by AutoDock Tools and other software. Lastly, Enzyme linked immunosorbent assay (Elisa) detection and Western blot analysis were utilized to further explore the possible mechanism of ZYTLF on PMOP.

**Results:**

With 129 target genes interacting with PMOP, 92 active compounds of ZYTLF corresponded to 243 targets, and 50 key genes were chosen. Network analysis revealed the top 10 active ingredients, such as quercetin and kaempferol and the top 50 key genes, such as ERα, p38 MAPK, p-AKT and TGF-β1. Enrichment analysis uncovered multiple signaling pathways, including estrogen signaling pathway, TNF signaling pathway, PI3K-Akt signaling pathway and MAPK signaling pathway. Furthermore, our finding of the foremost active compounds was tightly bound to the core proteins, which were verified by molecular docking analysis. Through experimental studies, we confirmed that the prescription of ZYTLF could ameliorate the OVX-induced bone loss, suppress the osteoclast activity and boost osteoblast ability through experimental studies.

**Conclusion:**

The potential mechanisms and therapeutic effects of ZYTLF against PMOP may be ascribed to inhibition of osteoclast activity, boost of osteoblast activity and enhancement of the expression of ERα.

**Supplementary Information:**

The online version contains supplementary material available at 10.1186/s13020-021-00503-5.

## Background

Postmenopausal osteoporosis (PMOP) is regarded as a severe chronic metabolic bone disease. There is a growing number of postmenopausal women around the world suffering from increased fragility and fracture susceptibility because of decreased ovarian function and estrogen level every year. At this stage of life, the bone resorption is faster than the bone building. Therefore, the incidence rate of osteoporosis steeps up in the population of women at menopausal age with plummeted estrogen level. Factors such as age, gender, genetics, reproductive status, calcium: phosphorus ratio, and exercise have certain impact on bone strength. Bone strength is consisted of bone mineral density (BMD) and bone quality. BMD is detected with dual energy absorptiometry (DXA) and declined by 30–40% at the age of 70s. However, there are many elderly patients who present with vertebral fractures but have bone density within the normal range in the ward. Bone quality deterioration is also closely associated with fracture [1, 2]. With characteristics of low bone mass and bone microstructure destruction in histological morphology, PMOP is associated with fractures due to decreased bone strength. As a common complication, osteoporotic fracture is one of the main culprits that leads to disability and death in postmenopausal women. More than 2 million osteoporosis-related fractures occur in American each year, and more than 70% of them occur in women. As the population ages, the cost of these fractures is estimated to exceed $25 billion by 2025 [3]. A currently published nationwide survey presented the prevalence of osteoporosis in China was 6.46% and 29.13% for men and women aged 50 years and older, respectively [4]. Another study constructed a state-transition microsimulation model and predicted the annual number of osteoporosis-related fractures in China would be 4.83 million by 2035 [5]. Osteoporosis and osteoporotic fracture have become a worldwide health problem, posing a huge burden both on individuals and society for its enormous cost and high risk of subsequent complications like pneumonia [6].

Hormone replacement therapy (HRT) in postmenopausal women enhances their estrogen levels and effectively reduces bone resorption, but its safety is still controversial [7]. A meta-analysis which included twenty-seven randomized controlled trials, indicated that alendronate had better efficacy on improving BMD and lower risk of adverse effect than raloxifene [8]. With high efficacy of inhibiting bone resorption, bisphosphonates are perceived as the first-line therapy for PMOP; however, its unclear short-and long-term safety also arouse public concerns. Therefore, limited options of clinically available treatments for PMOP highlight the urgent demand to develop alternative agents with better efficacy and safety. As one of the major modalities in complementary and alternative medicine, traditional Chinese medicine (TCM) has a long history in treating osteoporosis. It has low side effect, rich resources and remarkable efficacy. According to modern pharmacological experiments, various traditional Chinese herbs contain active ingredients against osteoporosis, and formulas of traditional Chinese herbs can treat osteoporosis in a more effective way than a single herb does [9, 10]. TCM, as an optional therapy, has got increasingly more attention.

With specific therapeutic activity and low side effect, ZYTLF has been prescribed for decades in Guangdong Province Hospital of Traditional Chinese Medicine to prevent and treat postmenopausal osteoporosis. Our previous study demonstrated that ZYTLF could effectively alleviate clinical symptoms in patients and increase their bone density by inhibiting bone resorption, reducing bone turnover and improving bone microstructure [11]. As a folk remedy, ZYTLF contains fourteen herbs: *Radix Rehmanniae Praeparatae* (Shu Di) as the monarch herb, *Ophiopogon japonicus* (Mai Dong), *Fructus Ligustri Lucidi* (Nv Zhen Zi), *Radix Angelicae Sinensis* (Dang Gui), *Radix Paeoniae Alba* (Bai Shao) as the minister herb, *Loranthus parasiticus* (Sang Ji Sheng), *Achyranthes bidentata* (Niu Xi), *Caulis Spatholobi* (Ji Xue Teng), *Zaocys* (Wu Shao She), *Scolopendra, Radix Astragali* (Huang Qi), *Saposhnikoviae Radix* (Fang Feng), *Rhizoma Atractylodis Macrocephalae* (Bai Zhu) as the assistant herb, *Radix Glycyrrhizae Preparata* (Gan Cao) as the guide herb. *Radix Rehmanniae Praeparatae* and its derivatives have been reported exerting bone protecting effect in the osteoporosis model by maintaining homeostasis between osteoclastogenesis and osteoblastogenesis [12]. *Ophiopogonin D*, which was isolated from the *Ophiopogon japonicus*, showed anti-osteoporosis effect via reducing reactive oxygen species in vivo [13]. Meanwhile, *Fructus Ligustri Lucidi* is proved to have the function of improving bone metabolism and bone quality in ovariectomized, growing, aged and diabetic rats through the regulation of OPG/RANKL/cathepsin K and other signaling pathways [14]. In addition, TCM formulas may exert complex synergistic or antagonistic effects to increase efficacy and decrease toxicity [15]. The mentioned effects provide a therapeutic rationale for a possible action of ZYTLF in promoting osteoclastogenesis and mitigating osteoblastogenesis. But there was no compelling evidence of its pharmacological mechanism on anti-osteoporosis.

Network pharmacology is an emerging technology, which integrates systems biology and bioinformatics and other emerging interdisciplinary disciplines to reveal the relationship between drugs and the body from the perspective of biological systems network. Li first applied network pharmacology to TCM and proposed the concepts of “TCM network pharmacology” and “network target” [16, 17]. In recent years, network pharmacology research has seen a blowout growth. Therefore, Li et al. recently issued a network pharmacology evaluation method guidance to standardize network pharmacology study [18]. Molecular simulation docking technology is an important technology in computer-aided drug research. It is mainly a theoretical simulation method to study the interaction between molecules and predict their binding patterns and affinity. It can clarify the mechanism of actions between the bioactive components of traditional Chinese medicine and the target at the molecular level [19].

**Fig. 1:**
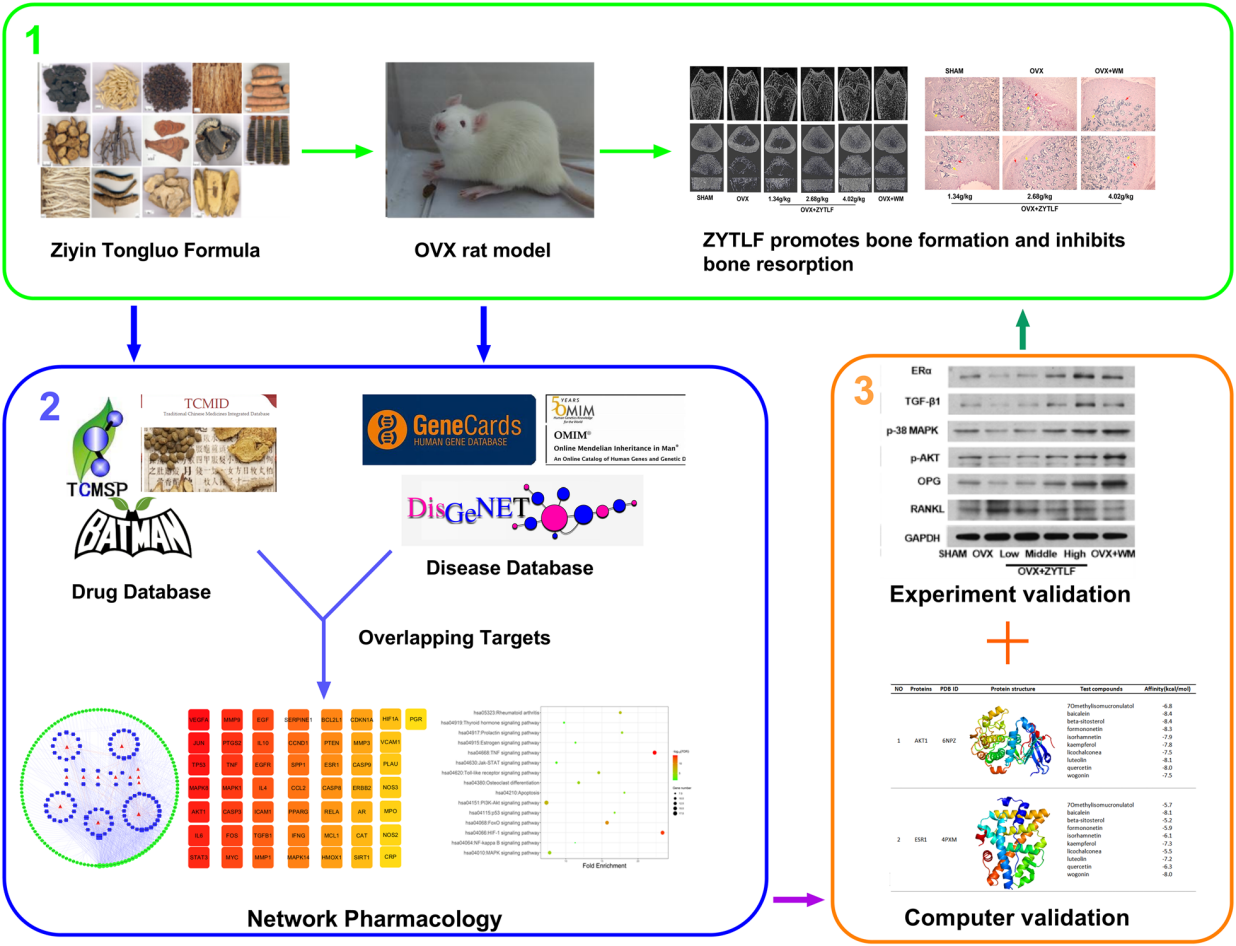
Scheme for the study of potential mechanism of ZYTLF in the treatment of PMOP via network pharmacology and ovariectomized rat model. In this study, OVX rat model was established and administrated with ZYTLF. The result showed that ZYTLF can significantly improve bone mineral density and bone microstructure of OVX rats, confirmed by ALP + TRACP staining that ZYTLF can promote osteogenic activity and inhibit osteoclast activity. Furthermore, the network pharmacology technique was applied to predict the active components and potential targets of ZYTLF against PMOP. Finally, molecular docking technology was utilized to perform preliminary validation computationally. WB analysis on bone tissue of OVX rats was used to validate the mechanism of ZYTLF against PMOP

In this work, network pharmacology was applied to mine the key ingredients, targets and signaling pathways of ZYTLF against PMOP. Moreover, molecular docking simulation was utilized to validate the stability of key proteins and corresponding compounds by detecting binding affinity. Furthermore, ovariectomized (OVX) rat models was established to confirmed the therapeutic effect of ZYTLF on PMOP and elucidate its preliminary mechanisms (Fig. 1).

## Methods

### ZYTLF preparation

The name and content of fourteen herbs in ZYTLF are shown in Table 1. The qualified granule ingredients of ZYTLF were purchased from Guangdong Province Hospital of Traditional Chinese Medicine Zhuhai Branch. Sixty grams of ZYTLF granules components were blended with 40 ml ultrapure water to make an ultimate density of 3 g/ml.

**Table 1:** Composition of ZYTLF

Pharmaceutical name	Botanical or zoological name	Chinese	Content (g)
*Radix Rehmanniae Praeparatae*	*Rehmannia glutinosa Libosch*	Shu Di	10
*Ophiopogon japonicus*	*Ophiopogonis Japonicum Tuber*	Mai Dong	10
*Achyranthes bidentata*	*Achyranthes Bidentata Radix*	Niu Xi	10
*Fructus Ligustri Lucidi*	*Ligustrum lucidum Ait *[fruit]	Nv Zhen Zi	10
*Radix Angelicae Sinensis*	*Angelica sinensis (Oliv.) Diels*	Dang Gui	10
*Radix Paeoniae Alba*	*Paeonia lactiflora Pall*	Bai Shao	10
*Loranthus parasiticus*	*Viscum Coloratum seu Loranthi Ramus*	Sang Ji sheng	15
*Radix Astragali*	*Astragalus mongholicus*	Huang Qi	10
*Zaocys*	*Ptyas dhumnades*	Wu Shao She	10
*Caulis Spatholobi*	*Spatholobus suberectus Dunn*	Ji Xue Teng	15
*Scolopendra*	*Scolopendra subspinipes mutilans*	Wu Gong	10
*Saposhnikoviae Radix*	*Ledebouriella Sesloides Radix*	Fang Feng	10
*Rhizoma Atractylodis Macrocephalae*	*Atractylodes macrocephala Koidz*	Bai Zhu	10
*Radix Glycyrrhizae Preparata*	*Glycyrrhiza uralensis Fisch*	Gan Cao	3

### Generation of OVX animal models

Experimental animal ethics panel of Guangdong Provincial Hospital of Traditional Chinese Medicine (License No. 2019044) approved the animal experiments. Experiments were performed in China Academy of Chinese Medical Sciences Guangdong Branch (License No. 00228297). Sixty three-month old female Sprague-Dawly (SD) rats, weighting 200–230 g, were purchased from the Laboratory Animal Center of Southern Medical University (License No. 44002100022874) and were allowed to acclimatize for one week before free access to food and space. All animal procedures complied with international ethical guidelines and the National Institutes of Health Guide concerning the Care and Use of Laboratory Animals. We tried our best to attenuate the number and suffering of the animals.

The rats received either sham operation (SHAM, n = 10) or bilateral ovariectomy (OVX, n = 50). All operations were performed with the application of anesthesia methods and sterile techniques as well as postoperative anti-infection and analgesia. Three months after ovariectomy, if the BMD of the rats were decreased significantly, the establishment of OVX animal model wound be considered to be successful.

### ZYTLF administration

Five groups of qualified rats were randomly divided (OVX, OVX + ZYTLF low dose, OVX + ZYTLF mid dose, OVX + ZYTLF high dose, OVX + WM (Western Medicine)). The rats in the SHAM group and OVX group were given distilled water by gavage, the OVX + ZYTLF Low/Middle/High dose group were administrated 1.34 g/kg, 2.68 g/kg, 4.02 g/kg per day respectively, and the OVX + WM group were treated with alendronate 10.51 mg/kg per week and calcitriol 0.075 μg/kg per day for 12 weeks. Rat intake dose was modified according to half, one or two times of the clinical human dose. The conversion obeys the Human-Rat Equivalent Dose Conversion Principle.

After the last 24 h of medicinal treatment, overdose of anesthesia was used to euthanize the animals. The rats were anesthetized after three months. Blood samples from the abdominal aorta were first centrifuged at 4000 rpm for 10 min. Then the serum was removed and the samples were stored at – 20 °C. Lumber vertebra body and bilateral femur and tibia were also removed as samples.

### Enzyme linked immunosorbent assay (Elisa) detection

The contents of estradiol (E2), alkaline phosphatase (ALP), procollagen I N-terminal propeptide (PINP), tartrate-resistant acid phosphatase (TRACP), type I collagen cross-linked Amino Terminal Peptide (NTX), type I collagen cross-linked carboxyl Terminal Peptide (CTX-1), calcium (Ca), phosphorus (P) in rat serum samples were confirmed by commercial ELISA kits based on manufacturer’s instructions. The ELISA kits were purchased from Nanjing Jiancheng Bioengineering Institute, Nanjing, China.

### Micro-CT bone analysis

Bone structural and mineral changes in rats were evaluated by micro-computed tomography (micro-CT) technique. A micro-CT imaging system (Bruker Skyscan 1172, Belgium) was applied to perform the CT scan and trabecular morphometric analysis under the guidance of manufacturer’s application notes. A spatial resolution of 9 μm (X-ray source 80 kV, 384 μA; 1 mm filter applied) was used to scan right femur samples. After that, CT images were reestablished by in-built software CT-vox and CTAn, respectively. With recommended order set, CTAn selected the trabecular region of interest in an unbiased batch. The aim of performing trabecular analysis was to quantify morphometric calculations and BMD.

The measured direct trabecular metric parameters of right femur were as follows: trabecular thickness (Tb.Th), bone volumetric fraction (BV/TV), trabecular number (Tb.N) as well as trabecular separation (Tb.Sp). Calculations of directly measured non-metric parameters were performed as well, including the trabecular bone pattern factor (Tb.Pf), an inverse assessment of trabecular connectivity and the structural model index (SMI), an estimate of the prevalence of plate-like or rod-like trabecular.

### Pathological observation

The left tibial tissue was acquired and fixed in 4% paraformaldehyde for 24 h and then decalcified for 2 weeks in 10% EDTA buffer (pH 7.0). The samples were dehydrated, embedded in paraffin and cut into slices (about 4 μm thick). Slices were processed with tartrate-resistant acid phosphatase (TRAP)-staining and alkaline phosphatase (ALP)-staining respectively, aiming to detect the number of osteoclast and osteoblast. The sections were visualized by using an optical microscope (Olympus, shanghai, China) and then photographed. The cytoplasm of osteoclasts was wine-red and the cytoplasm of osteoblasts showed grayish-black granules after ALP + TRACP staining.

### Network pharmacology analysis

#### Database preparation

Fourteen herbs of ZYTLF were in inputted into the Traditional Chinese Medicine Systems Pharmacology Database and Analysis Platform (TCMSP http://tcmspw.com/tcmsp.php) [20]. BAT-MAN TCM (http://bionet.ncpsb.org/batman-tcm/) [21] and Traditional Chinese Medicine Information Database (TCMID, http://www.megabionet.org/tcmid/) [22] in sequence to find certain or potential corresponding compounds. Oral bioavailability (OB) and drug-likeness (DL) were set as standards for screening active compounds. Compounds that were considered as biologically active ingredients shall meet the standard of OB ≥ 30% and DL ≥ 0.18. Since *Radix Glycyrrhizae Preparata* serves as the guide herb in ZYTLF with a small amount, greater bias was avoided by setting the standard as OB ≥ 60% and DL ≥ 0.36. The targets of ZYTLF were collected by TCMSP analysis platform and then were transferred to standard protein name in Uniprot database (https://www.Uniprot.org), setting organisms as Human [23].

“Postmenopausal” and “Osteoporosis” were the key words to gather PMOP connected genes from the three following online databases: Genecards (http://www.genecards.org) [24], OMIM (http://omim.org/) [25], and DisGenet (https://www.disgenet.org/) [26]. Overlapping target genes that could be potential targets for ZYTLF against PMOP were later obtained from ZYTLF target genes and PMOP-related genes.

#### Network analysis

The network model of “herb-compound-overlapping gene” was built by importing the overlapping target genes, their corresponding active components and herbs into Cytoscape 3.7.1 [27]. CytoNCA plug-in [28] was applied to perform network topology analysis. Key nodes in the network were screened in light of the Degree Centrality (DC) and Between Centrality (BC). The higher the node's degree value was, the more important it was in the network.

Construction of protein–protein interaction (PPI) network of overlapping proteins was completed through setting the condition of data analysis mode as “Multiple proteins”, the type as “Homo sapiens” (human), and the minimum mutual threshold as “high confidence (0.700)” in The STRING database (http://string-db.org/, ver.11.0) [29]. The other parameters were unaltered. The data of PPI network was obtained and then inputted into Cytoscape 3.7.1. Core genes were screened out by network analysis conducted with The MCC algorithm in the CytoHubba plug-in.

The Database Visualization and Integrated Discovery system (DAVID, https://david.ncifcrf.gov/) was utilized to conduct enrichment analysis of Gene Ontology (GO, http://www.geneontology.org/) and Kyoto Encyclopedia of Genes and Genomes (KEGG, http://www.genome.jp/kegg/) [30, 31], setting race “Homo sapiens”. Advanced bubble diagrams were drawn by R software.

### Molecular docking simulation

The binding efficiency of the overlapping proteins and major active components in ZYTLF were evaluated by computer simulation docking technology. The SDF structure of top 10 core compounds were collected in the network from the PubChem database (https://pubchem.ncbi.nlm.nih.gov/). Procession and transformation of the 2D structure into PDB format were accomplished by PyMOL, and they were saved in PDBQT format as docking ligands. Concurrently, the collection of all the crystal structures of the key proteins from the RCSB protein data bank (PDB, http://www.pdb.org/) and the selection of those with distinctive ligands and comparatively higher resolution were done. AutoDock Tools was used to take away the water molecules, isolate proteins and reserve them as receptors. The receptors and ligands were processed with PyMOL and Auto Dock and then docked through Vina.

### Western blot analysis

Radioimmunoprecipitation assay (RIPA) buffer that contained Halt Phosphatase Inhibitor Cocktail (Thermo Scientific, USA) were ground with right tibia samples, which were corrected based on the results of the bicinchoninic acid assay (BCA). 10% SDS-PAGE separated 30 µg protein. Then they were transferred to polyvinylidene fluoride (PVDF) membranes (Millipore, USA). In order to maximize the protein loading, membranes were air-dried and reactivated in methyl alcohol. According to the purpose of the experiment, they were blocked with 5% nonfat milk powder at room temperature, and incubated with primary antibody, including ERα (1:1000, Abcam, Cambridge, UN), TGF-β1 (1:1000, Cambridge, UN), p38 MAPK (1:2000, Cambridge, UN), p-AKT (1:1000, Cambridge, UN), and GAPDH (1:1000, Cambridge, UN) at 4 °C overnight. The nitrocellulose membranes were washed three times with PBST and incubated with goat anti-rabbit IgG antibody (1:15,000, Zs-BIO, Shanghai, China) at room temperature for 2 h. After these procedures, they were rinsed three times and scanned for optimal density value of the target protein unit by improved Journal Pre-proof chemiluminescence analysis (Thermo, MA, USA).

### Statistical analysis

All statistical analyses of this paper were performed by GraphPad Prism 8.0.2 (San Diego, USA). The data were represented by means ± standard deviation (SD). The nonparametric Kruskal–Wallis method was used for the data without Gaussian distribution based on Shapiro–Wilk test, followed by Dunn’s multiple comparisons test. One-way ANOVA analysis was used for the data with Gaussian distribution and equal SDs, followed by Dunnett’s multiple comparisons test. Brown-Forsythe ANOVA test was used for the data with Gaussian distribution and unequal SDs, followed by Dunnett’s T3 multiple comparisons test. Statistical analyses were conducted to confirm the differences between test groups and nominated control group, with the significance level at P < 0.05.

## Results

### Effects of ZYTLF on serum indicators in OVX rat

**Fig. 2:**
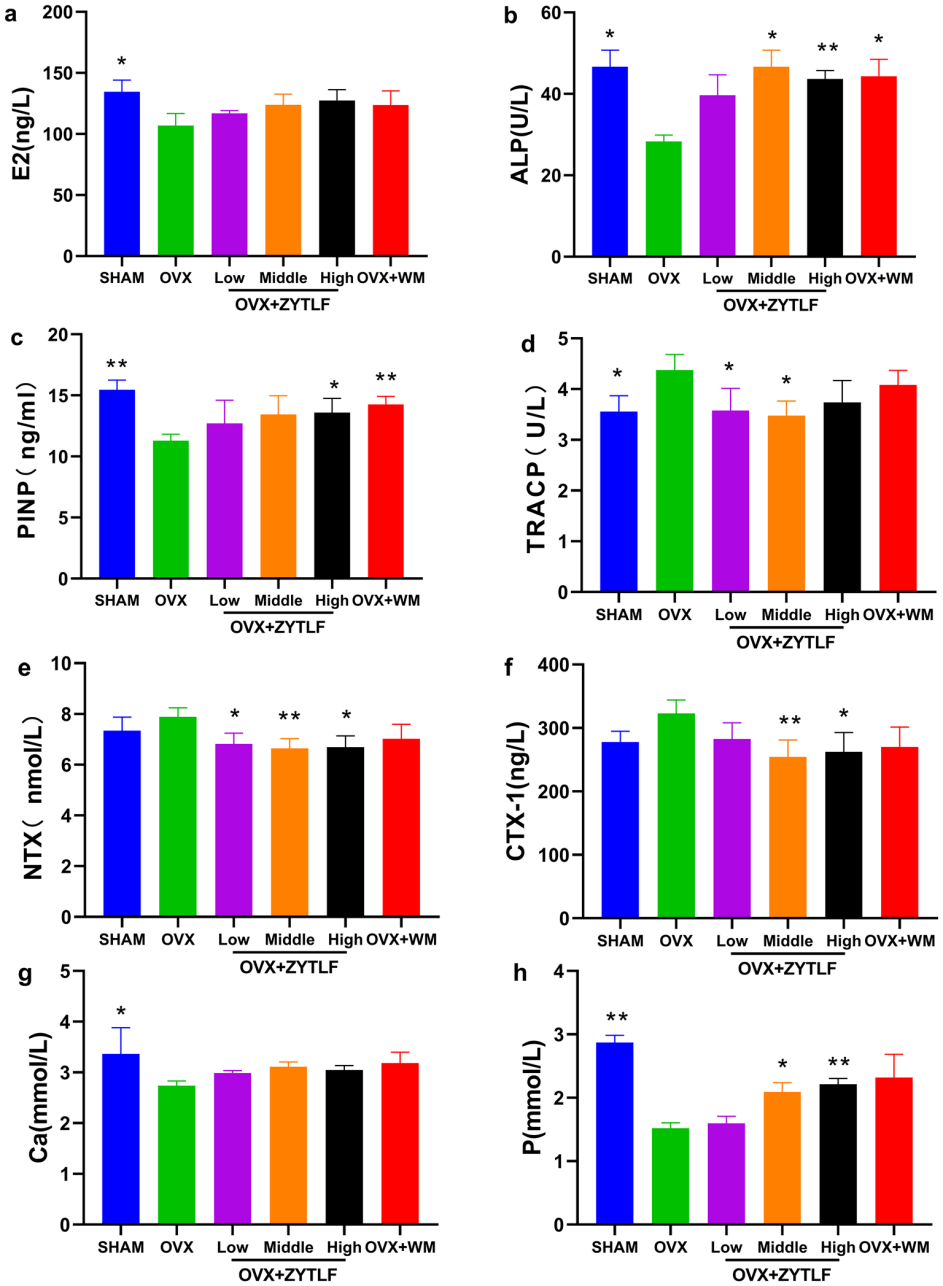
Effects of ZYTLF on serum indicators in OVX rat. **a** estradiol (E2), **b** alkaline phosphatase (ALP), **c** procollagen I N-terminal propeptide (PINP), **d** tartrate-resistant acid phosphatase (TRACP), **e** type I collagen cross-linked Amino Terminal Peptide (NTX), **f** type I collagen cross-linked carboxyl Terminal Peptide (CTX-1), **g** calcium (Ca), **h** phosphorus (P). The statistics with Gaussian distribution were shown as the mean ± SD (n = 9 rats per group). Low: 1.34 g/kg; Middle: 2.68 g/kg; High: 4.02 g/kg; Western Medicine: alendronate 10.51 mg/kg per week and calcitriol 0.075 μg/kg per day. *p < 0.05, **p < 0.01, when compared with OVX group

To determine the threptic effects of ZYTLF on OVX rats, we collected serum from experimental rats after treatment. Related indicators in serum were detected by ELISA, which the result of were shown in Fig. 2. The rats in OVX group showed lower level of E2 (p < 0.05, Fig. 2a), ALP (p < 0.01, Fig. 2b), PINP (p < 0.01, Fig. 2c), Ca (p < 0.05, Fig. 2g), P (p < 0.01, Fig. 2h) and higher level of TRACP (p < 0.05, Fig. 2d) compared with the sham group. We found that ZYTLF did not significantly change the levels of E2. However, it significantly increased the level of ALP (p < 0.05), PINP (p < 0.05) and P (p < 0.05) and inhibited the level of TRACP (p < 0.05), NTX (p < 0.05, Fig. 2e) and CTX-1 (p < 0.01, Fig. 2f) in comparison with the OVX group. ALP, PINP are considered as indicators of osteogenic activity, while TRACP, NTX and CTX-1 are deemed as indicators of osteoclastic activity. There is no significant difference in the level of Ca between ZYTLF treatment group and OVX group (p > 0.05).

### Micro-CT evaluation

**Fig. 3:**
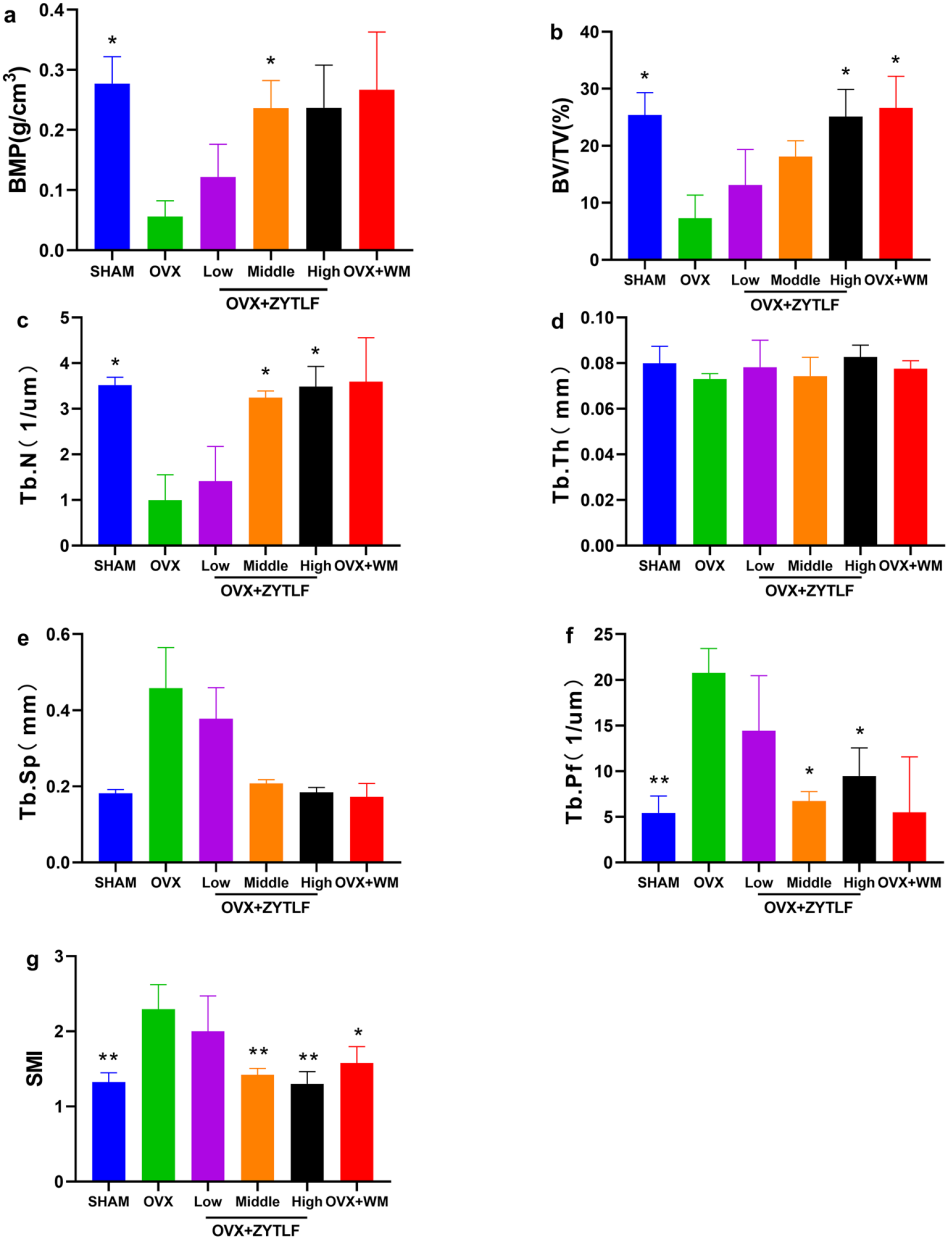
The effect of ZYTLF on trabecular bone parameters in the right distal femur of OVX rats. Parameters assessed: **a** bone mineral density (BMD); **b** bone volume fraction (BV/TV); **c** trabecular number (Tb.N); **d** trabecular thickness (Tb.Th); **e** trabecular separation (Tb.Sp); **f** trabecular bone pattern factor (Tb.Pf); **g** structure model index (SMI). The results were shown as the mean ± SD. Low: 1.34 g/kg; Middle: 2.68 g/kg; High: 4.02 g/kg; Western Medicine: alendronate 10.51 mg/kg per week and calcitriol 0.075 μg/kg per day. *p < 0.05, **p < 0.01 compared with OVX group.

To further determined the effect of ZYTLF on experimental animals, Micro-CT evaluation was performed on right femur. As shown in Fig. 3a, compared with the sham group, the BMD in the OVX group considerably decreased (p < 0.05). Alendronate and calcitriol elevated the BMD, as ZYTLF did. Middle dose of ZYTLF (p < 0.05) significantly elevated BMD compared with those in the untreated OVX rats. As for bone micro structure, SMI (p < 0.05, Fig. 3g) and Tb.Pf (p < 0.01, Fig. 3f) were noticeably more prominent in the OVX group, while BV/TV (p < 0.05, Fig. 3b) and Tb.N (p < 0.05, Fig. 3c) were markedly lower than those parameters in the SHAM group. In the middle and high dose of ZYTLF groups, Tb.N were significantly increased compared with those of the OVX group, while SMI was observably reduced in the middle and high dose of ZYTLF groups. Nevertheless, on BV/TV, Tb.N and Tb.Pf from OVX, 1.34 g/kg ZYTLF group represented weaker rescue effect in comparison with sham group. There is no significant different in the Tb.Th and Tb.Sp (p > 0.05, Fig. 3e) between ZYTLF treatment group and OVX group (p > 0.05, Fig. 3d).

**Fig. 4:**
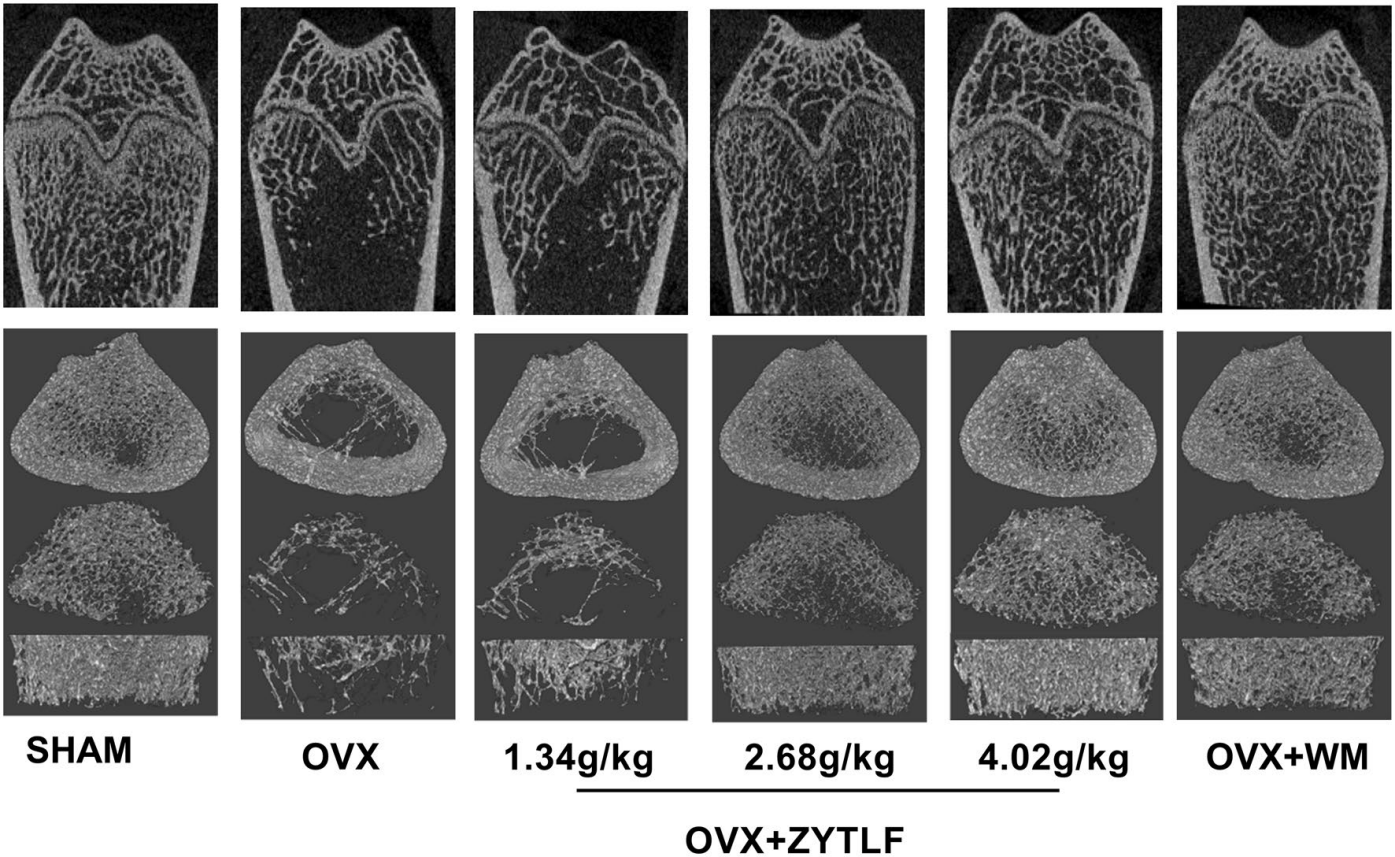
Micro-CT scan of the right distal femur. Representative plain scan images of the distal femur were shown (sagittal and transverse) in SHAM, OVX, OVX + ZYTLF (1.34 g/kg), OVX + ZYTLF (2.68 g/kg), OVX + ZYTLF (4.02 g/kg), OVX + WM (Western Medicine) respectively

The micro-CT plain scan (sagittal and transverse) of the distal femoral bone were shown in Fig. 4. Though according to all transverse and sagittal images, there’s no significant loss of cortical bone, trabecular bone had less spreading, thinning structure and dilated interval space in comparison with sham group. Consistent with BMD results, OVX rats reduced remarkably in trabecula. The middle and high dose of ZYTLF and western medicine markedly prevented bone mineral loss in trabecular bone from OVX.

### Pathological observation of the tibia

**Fig. 5:**
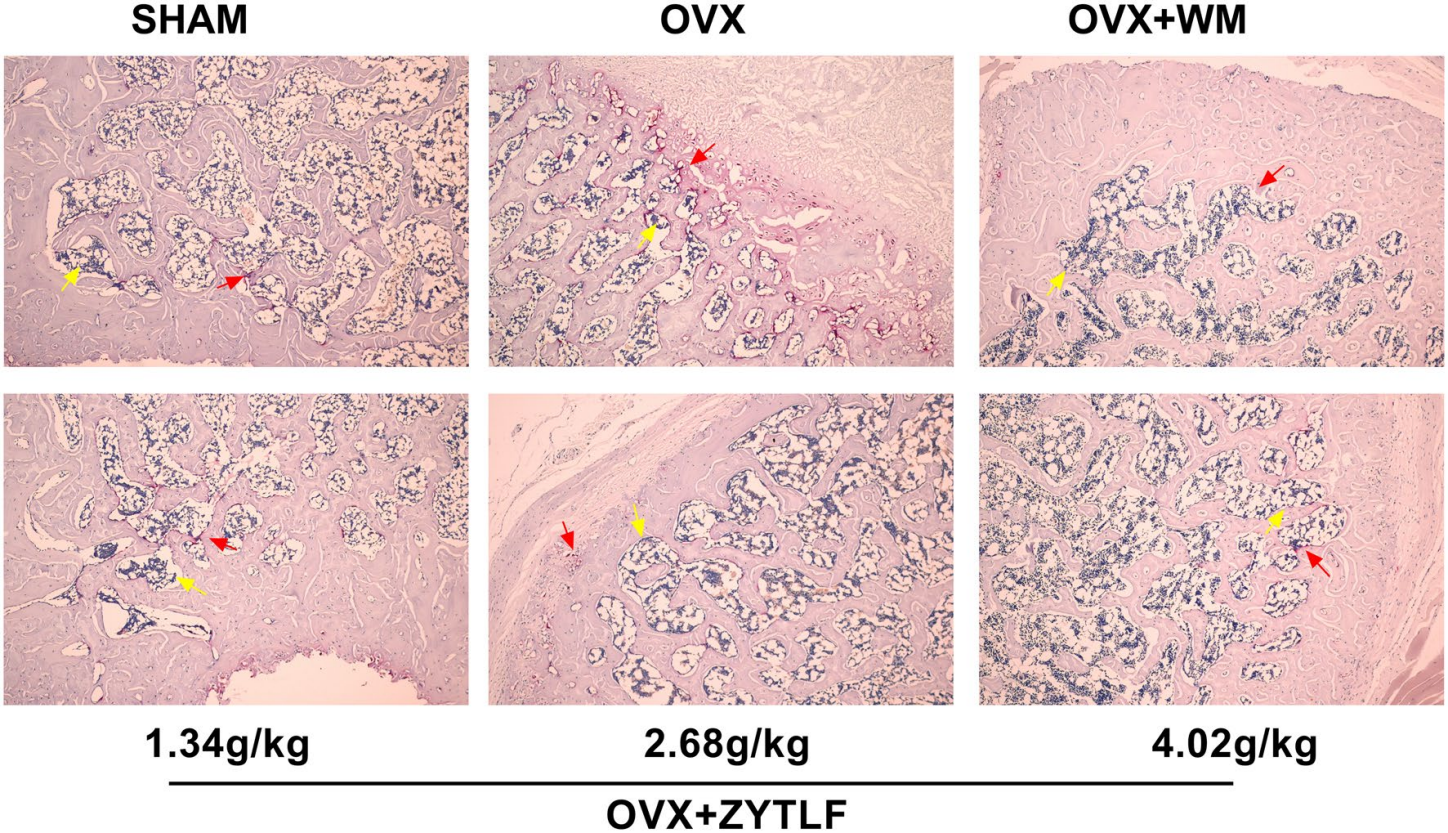
ZYTLF inhibited the formation of osteoclast in OVX rats. After treating, ALP + TRACP staining were performed and the results were shown in above (10×). The cytoplasm of osteoclasts is wine-red and the cytoplasm of osteoblasts shows grayish-black granules, noted by red and yellow arrow respectively

To verified the effect of ZYTLF on the activity of osteoclast and osteoblast, ALP + TRACP staining was conducted on bone biopsy. Figure 5 showed histological micrographs of the rat tibias. Osteoclast numbers, osteoblast numbers and morphology were normal in the tibia of the sham ground. The number of osteoclasts increased in the OVX ground but the number of osteoblasts declined. In the contrast, the number of osteoclast and osteoblast in the OVX + ZYTLF grounds were normal as the level of the sham ground.

### Active ingredients of ZYTLF and overlapping genes

**Fig. 6:**
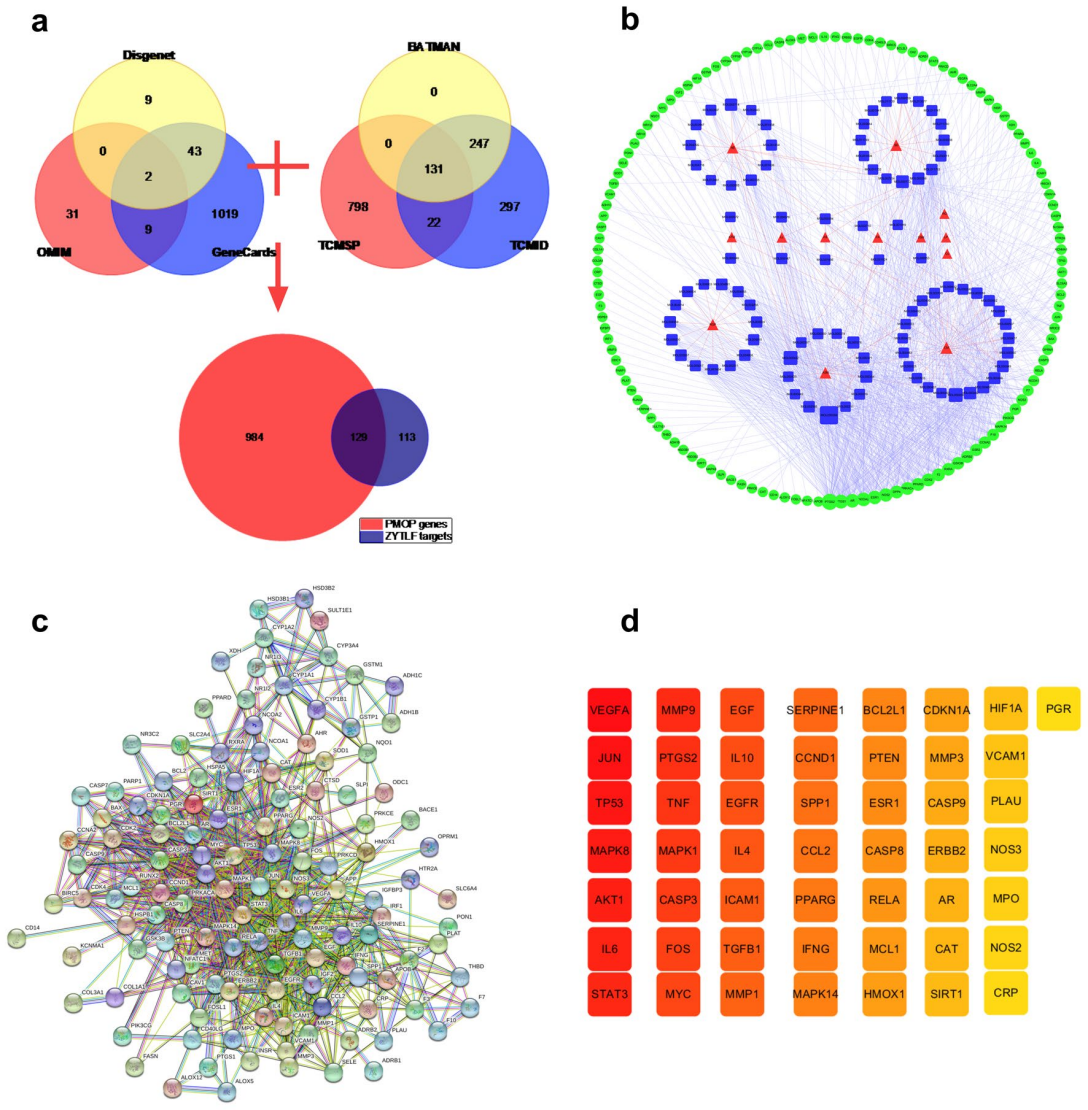
Hub proteins of ZYTLF were determined by network pharmacology technology. **a** Venn diagram: 129 overlapping genes were selected as potential targets for further study. **b** The herb-compound-overlapping gene network: the red triangle represents traditional Chinese herbs of ZYTLF, blue square represents compounds, and green circle represent overlapping target genes. Lines represent relationship between nodes. And the larger of nodes, the higher degree of constituent. **c** Protein–protein interaction (PPI) network of 129 overlapping proteins. **d** 50 hub proteins: after computing the overlapping proteins, hub proteins were screened. The color of the node changed from light yellow to dark red, indicating that the higher the MCC value was, the more significant the role it played in the network

A total of 1738 compounds were obtained from TSMSP platform, BAT-MAN TCM and TCMID database, among which 92 met the requirement were regarded as active components. Additional file 1: Table S1 displayed the elaborated data of active components of ZYTLF. There were totally 242 corresponding target genes for these active components. After removing duplication, totally 1113 PMOP related genes were collected From Genecard, Disgenet and OMIM databases. Compared target genes of ZYTLF with PMOP-related genes, 129 overlapping target genes were collected altogether (Fig. 6a). Details about ZYTLF-genes, PMOP-genes, and ZYTLF-PMOP overlapping target genes were presented in Additional file 2: Table S2.

### Network analysis

A network of “herb-compound-overlapping gene” was established (Fig. 6b). Additional file 3: Table S3 showed the detailed information of the network with 231 nodes and 1072 edges. According to topology analysis of the network, active ingredients such as quercetin, kaempferol, luteolin, scutellarein and formononetin had higher degrees, which also played a vital part within the network. After setting the relevant parameters, intersecting genes were imported into the STRING database to build a PPI network. The network represented the relevant details of the genes and the interaction association (Fig. 6c). Then the PPI network information was visually handled and analyzed by Cytoscape 3.7.1 software. The selection of top 50 key genes was based on the MCC algorithm of the CytoHubba plug-in (Fig. 6d).

### GO and KEGG enrichment analysis

**Fig. 7:**
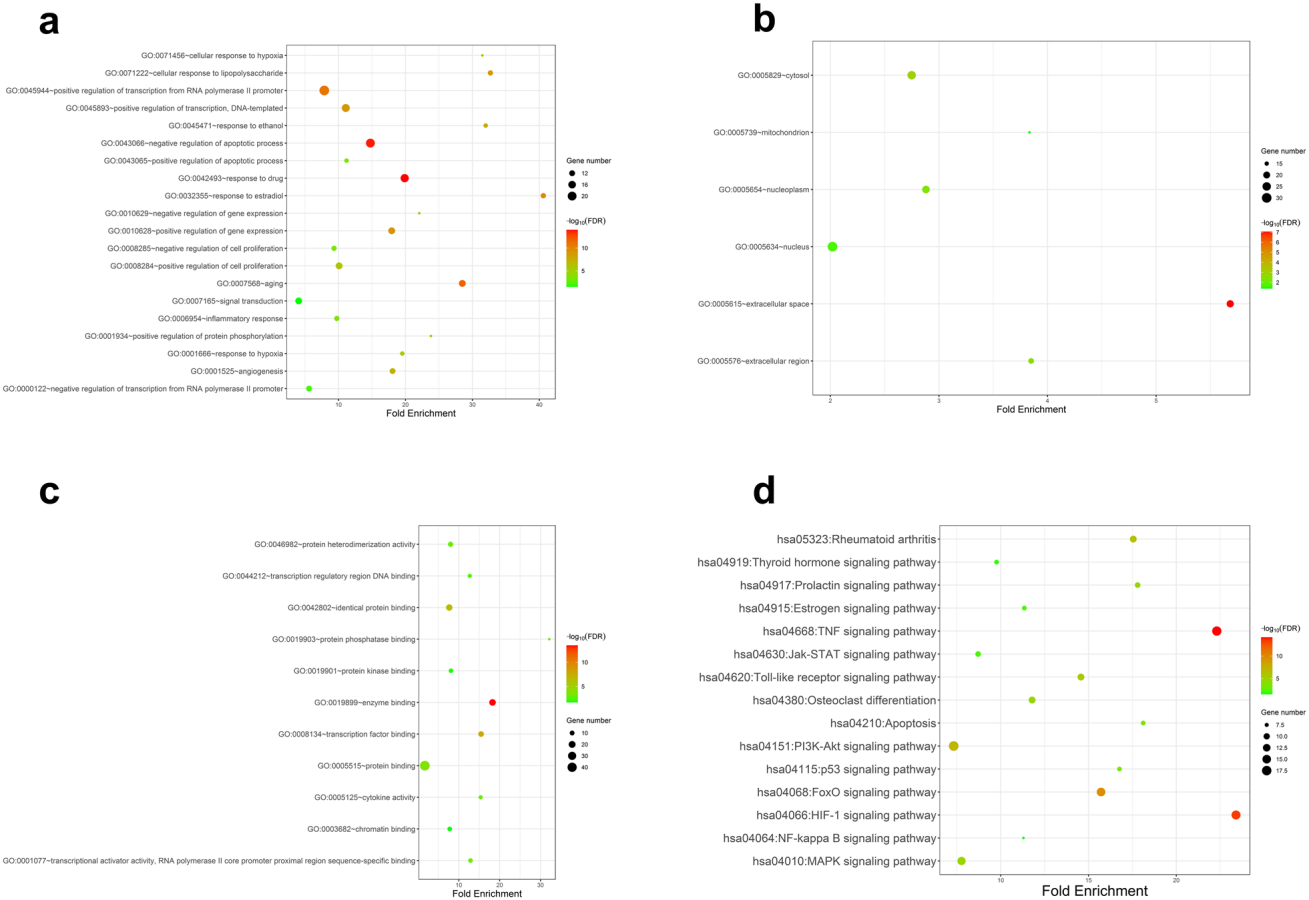
GO analysis and KEGG pathway enrichment analysis of Key gene. In the bubble diagrams above (**a**–**d**), the ordinate represents the names of BP, CC, MF terms and pathways, respectively, and the abscissa represents the degree of enrichment. The smaller the FDR is, the higher the importance of enrichment, and the redder the color on the diagram

Performance of GO enrichment and KEGG pathway enrichment analysis on 50 core genes and extraction of significant enrichment results (FDR < 0.05) were done by DAVID database. Totally, 62 GO-BP terms, 6 GO-CC terms, 11 GO-MF terms and 59 terms on the KEEG pathway were obtained. The enrichment results showed that the main biological processes were positive transcriptional regulation signals of RNA polymerase II promoter, negative regulation of apoptosis, positive regulation of transcription using DNA as a template, aging, positive regulation of gene expression, etc. Main cell components such as nucleus, cytoplasm and extracellular space were the major components of biological processes. Major regulated molecular functions were binding of enzyme, transcription factor and protein binding (Fig. 7a–c). PMOP is a disease with complicated pathogenesis, which involves the formation and apoptosis of osteoblasts and osteoclasts. Based on the results above, we carefully speculated that the ZYTLF acted against PMOP by affecting intracellular enzyme binding, protein binding, transcription factor and then regulating apoptosis and aging process, and gene expression. Fifteen signal pathways associated with PMOP were markedly enriched by KEGG analysis (Fig. 7d), including estrogen signaling pathway, TNF signaling pathway, PI3K-Akt signaling pathway, MAPK signaling pathway. Additional file 4: Table S4 elucidated the results and details on the GO terms and pathways.

### The core compounds in ZYTLF are docked with key proteins

Whether the top 10 compounds were essential in regulating the 50 key proteins was verified by molecular docking simulation. A stable structure wound be formed when the ligand bound to one or more amino acid residues in the active site (also called active pocket) of the receptor and took part in the process such as conformation change and energy complementation to combine with the receptor. This research illustrated that the top 10 compounds had sturdy association with 50 core proteins, such as AKT1 (PDB id: 6npz), MAPK14 (PDB id: 6sfo), ERα (PDB id: 4pxm), TNF (PDB id: 3m2w), TGF-β1 (PDB id: 6om2) and PTGS2 (PDB id: 5f19). These compounds were of great importance based on the result of network pharmacology (Table 2). The ligands and receptors were considered to be able to form stable compounds when the binding energy was less than − 5 kcal/mol. Details of the binding energies of the different compounds were shown in Additional file 5: Table S5.

**Table 2:** Information of key proteins and compounds by molecular docking

No	Proteins	PDB ID	Protein structure	Test compounds	Affinity(kcal/mol)
1	AKT1	6NPZ		7Omethylisomucronulatol	− 6.8
				Baicalein	− 8.4
				Beta-sitosterol	− 8.4
				Formononetin	− 8.3
				Isorhamnetin	− 7.9
				Kaempferol	− 7.8
				Licochalconea	− 7.5
				Luteolin	− 8.1
				Quercetin	− 8.0
				Wogonin	− 7.5
2	ESR1	4PXM		7Omethylisomucronulatol	− 5.7
				Baicalein	− 8.1
				Beta-sitosterol	− 5.2
				Formononetin	− 5.9
				Isorhamnetin	− 6.1
				Kaempferol	− 7.3
				Licochalconea	− 5.5
				Luteolin	− 7.2
				Quercetin	− 6.3
				Wogonin	− 8.0
3	MAPK14	6SFO		7Omethylisomucronulatol	− 7.4
				Baicalein	− 9.6
				Beta-sitosterol	− 7.4
				Formononetin	− 9.3
				Isorhamnetin	− 8.7
				Kaempferol	− 9.2
				Licochalconea	− 8.3
				Luteolin	− 9.3
				Quercetin	− 9.3
				Wogonin	− 8.9
4	PTGS2	5F19		7Omethylisomucronulatol	− 7.5
				Baicalein	− 9.0
				Beta-sitosterol	− 7.3
				Formononetin	− 8.4
				Isorhamnetin	− 9.2
				Kaempferol	− 8.1
				Licochalconea	− 8.9
				Luteolin	− 8.9
				Quercetin	− 8.7
				Wogonin	− 8.4
5	TNF-α	3M2W		7Omethylisomucronulatol	− 7.7
				Baicalein	− 9.4
				Beta-sitosterol	− 9.2
				Formononetin	− 9.0
				Isorhamnetin	− 9.1
				Kaempferol	− 8.9
				Licochalconea	− 8.7
				Luteolin	− 9.5
				Quercetin	− 9.2
				Wogonin	− 9.2
6	TGFβ1	5VQP		7Omethylisomucronulatol	− 8.3
				Baicalein	− 7.6
				Beta-sitosterol	− 7.9
				Formononetin	− 9.3
				Isorhamnetin	− 8.5
				Kaempferol	− 8.4
				Licochalconea	− 9.0
				Luteolin	− 7.3
				Quercetin	− 9.4
				Wogonin	− 8.5

Partial result of molecular simulation docking result of binding of key proteins and active components. When the binding energy is less than − 5 kcal/mol, the ligands and receptors are considered to be able to form stable compounds

### ZYTLF modulated the expression of ERα, p38 MAPK, p-AKT, TGF-β1, OPG, RANKL and OPG/RANKL ratio

**Fig. 8:**
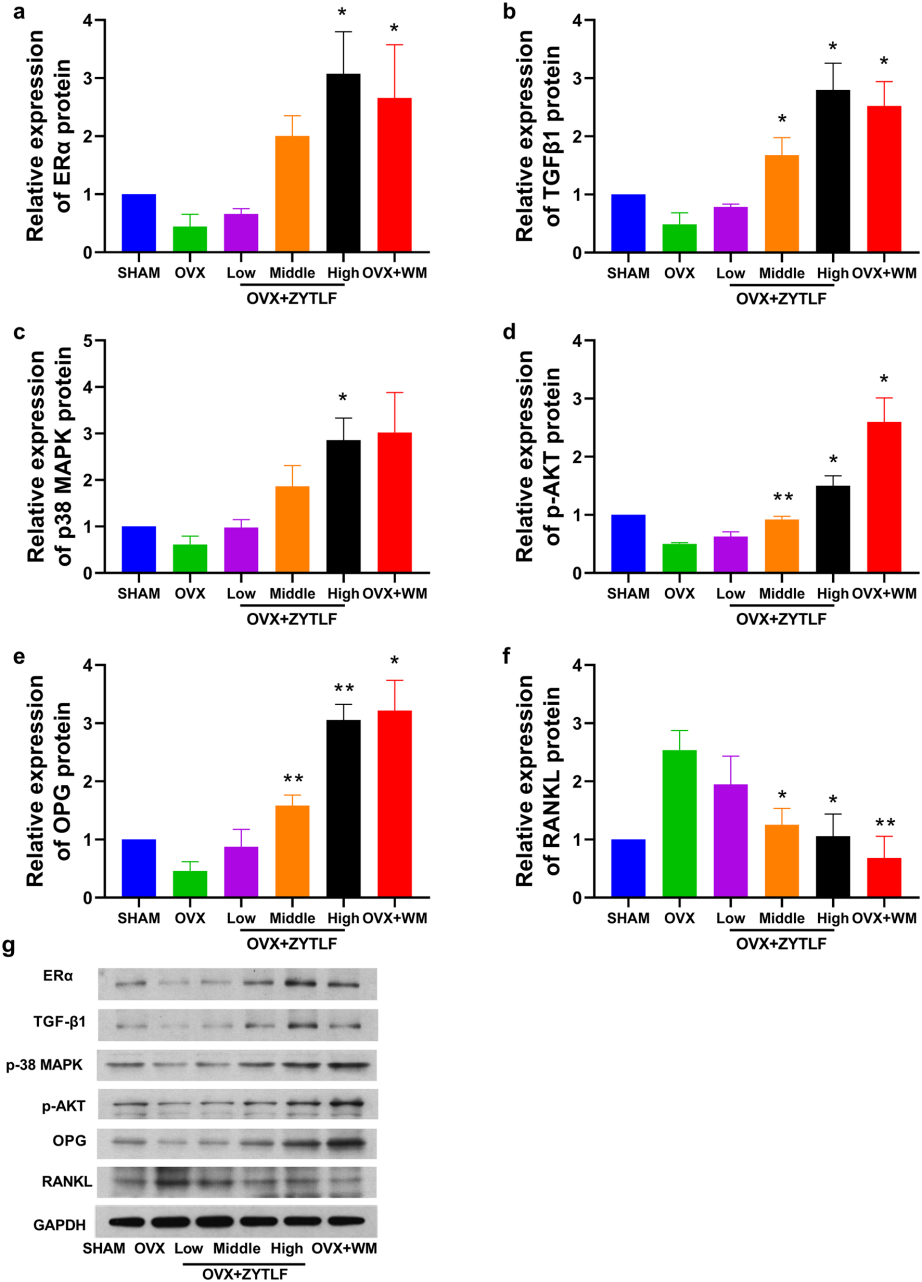
Effects of ZYTLF on expressions of key proteins. After treatment, the protein ERα (**a**), p38 MAPK (**b**), p-AKT (**c**), TGF-β1 (**d**), OPG (**e**), RANKL (**f**) expression of tibia in rats were measured by western blotting. The quantified amount of proteins was calibrated with the amount of GAPDH and then compared with the value of sham group. The statistics with Gaussian distribution were shown as the mean ± SD. Low: 1.34 g/kg; Middle: 2.68 g/kg; High: 4.02 g/kg; Western Medicine: alendronate 10.51 mg/kg per week and calcitriol 0.075 μg/kg per day. *p < 0.05, **p < 0.01 compared with OVX group

To determine the mechanism in ZYTLF treated PMOP, expression of six key proteins in bone tissues after ZYTLF treatment were examined (Fig. 8). ERα, TGF-β1, p38 MAPK, p-AKT, OPG protein level in bone tissue was dramatically elevated by ZYTLF treatment in contrast with OVX group in a dose-dependent manner. Moreover, RANKL protein level was suppressed markedly in ZYTLF groups.

## Discussion

PMOP is a serve public issue worldwide that remarkably increased the risk of fracture in postmenopausal women. Due to its complex pathological mechanism, the current treatments for PMOP somewhat feeble. TCM, as a complicated ancient Chinese traditional therapy, has received more and more attention for its efficacy. As one of Chinese prescriptions, ZYTLF have received clinical effect and be populated among patients.

In this study, ovariectomized rat model was successfully established and then the estrogen level decreased suddenly and dramatically. Estrogen deficiency boosts bone resorption by stimulating osteoclast formation and lifespan. Under this condition, osteoblast formation is also stimulated, but this increased bone turnover shifts the bone homeostasis toward bone resorption, resulting in rapid trabecular bone loss and an increased risk of skeletal fracture. The SD rats undergone ovariectomy without treatment showed lower BMD and worse bone micro structure.

In this study, we found that ZYTLF treatment inhibited the bone loss in OVX rats. The BMD of OVX rats was improved by low(p > 0.05), middle(p < 0.05) and high(p > 0.05) dose of ZYTLF. Moreover, ZYTLF showed similar effect to alendronate on trabecular microstructure indexes. The results indicated that ZYTLF could mitigate microstructure degeneration in OVX rats and preserve thicker, interwoven and plate-like trabecular bone. These observations demonstrated that ZYTLF could ameliorates trabecular bone resorption in OVX induced rat model with high turnover bone metabolism. Furthermore, our bone pathological analysis with ALP + TRACP staining and detection of bone metabolic markers confirmed that ZYTLF could ameliorate the bone phenotype of OVX rats, reduce the osteoclast formation and increase osteoblast differentiation. Many TCM therapy has an effect on promoting OB generation and inhibiting OC differentiation, as ZYTLF does. However, the specific mechanism of ZYTLF on osteoclastogenesis and osteoblastogenesis is still uncertain.

Preliminary determination of the pharmacological compounds and complex molecular mechanisms of ZYTLF on PMOP were done by molecular docking technology and network pharmacology. Network pharmacology is a systematic approach employed to learn the complexities among compounds, targets, diseases and biosystems. It coincides with the holistic and systemic views of TCM theory [32]. Currently, the pharmacological action, safety and sophisticated molecular mechanisms of TCM are mostly investigated by network pharmacology. The results from network pharmacology were further verified by the computer simulation docking technology through evaluation of the binding efficiency of the overlapping proteins and main active components in ZYTLF. Through the screening of active ingredients and analysis of compound-target network, quercetin and kaempferol were considered as the main compounds of ZYTLF that exerted pharmacological effect on PMOP. With the highest degree value in the network, quercetin is a representative flavonoid compound. It has various pharmacological effects, such as anti-infection, anti-cancer, anti-free-radical, and cardiovascular protection [33–36]. Li found that possibly by up-regulating gene expression of ALP and inhibiting signaling pathways of JNK, ERK, and p38 MAPK, quercetin could ameliorate osteoporosis symptoms in ovariectomized rats [37]. Kaempferol has also been proved of protecting bones on ovariectomized rats [38], possibly through estrogen receptor, MAPK, NF-κB and other signaling pathways [39].

Additional, network-based algorithm was utilized on key proteins to understand the gene ontology (GO) and KEGG pathways in the treatment of ZYTLF. From the result, we assumed that ZYTLF might manipulated multiple biological processes and molecular functions, consisted with the complexed mechanisms of PMOP. The data revealed 50 putative targets were involved in pharmacological action of ZYTLF on PMOP, in which ERα, p38 MAPK, p-AKT, TGF-β1 were considered as hub proteins. Moreover, 15 KEGG pathways were observably enriched by KEGG analysis, including estrogen signaling pathway, TNF signaling pathway, PI3K-AKT signaling pathway, MAPK signaling pathway, which were closely bound with the development and progression of PMOP. Estrogen bound with the estrogen receptor in osteoblasts and osteoclasts to act on the OPG/RANK/RANKL signaling pathway, which further propelled secretion of OPG, down-regulated the expression of RANKL, and inhibited the formation of osteoclasts [40]. It is widely acknowledged that the falling level of estrogen in postmenopausal women can stimulate the immune system to supply an oversized amount of osteoclastogenic factors, which later activates related signaling pathways and further aggravates bone loss [39]. According to in-vitro experiments, through NF-κB and PI3K/Akt signaling pathways, TNF-αand RANKL concertedly improve bone resorption of osteoclasts [41].

It is generally considered that estrogen deficiency is the major cause of the occurrence and development of PMOP, so the current research mainly focuses on how to elevate estrogen level. Interestingly, this research found that ZYTLF did not significantly boost estrogen level, but reduce bone loss to normal bone homeostasis. Through western-blot analysis, we found that ZYTLF treatment increased the expression of ERα protein in bone sample. ERα has been proved to play a critical role in bone metabolism. The expression of ERα in bone tissues is estrogen-dependent, and the transcription and translation of ERα are inhibited after the level of estrogen is declined [42]. On the other hand, after the combination of estrogen and estrogen receptor, it could also regulate the expression of various target genes through the estrogen signaling pathway, so that downstream PI3K/Akt, MAPK, WNT and other signaling pathways could be activated to enhance the proliferation and differentiation of osteoblasts [43, 44]. Our study suggested that excessive expression of ERαmay activate the downstream proteins, including p-AKT, p38-MAPK, TGF-β1, OPG. By increasing estrogen receptor expression, rather than raising estrogen level, this may be a new idea of treatment of PMOP in the future.

Compare our study with the Network pharmacology evaluation method guidance [18]. There are also several limitations about this study. Firstly, we strictly searched and screened the active components from a variety of databases, but there are many small compounds of ZYTLF, which interact with each other, so the results may be biased and lack of experimental verification. Secondly, molecular docking technology and animal models were applied to validate the predicted results, and the anti-osteoporosis mechanism of ZYTLF was revealed. Due to the limited funding, there may be a lack of multiple verification, including experiments at gene level or with cell model.

## Conclusion

In summary, our findings illuminated that ZYTLF could ameliorate the OVX-induced bone loss, suppress the osteoclast activity and boost osteoblast ability. Moreover, the key compounds and target proteins were mined, such as quercetin, kaempferol and ERα, p38 MAPK, p-AKT and TGF-β1, which were considered as the materials of ZYTLF in treatment of PMOP. In addition, multiple signaling pathways, including estrogen, MAPK, PI3K/Akt and OPG/RANK/RANKL signaling pathways, involved the treatment mechanisms. Consequently, this study provides rational treatment of ZYTLF on postmenopausal osteoporosis with experimental evidence and preliminarily indicated its mechanism.

## Supplementary Information

**Additional file 1: Table S1.** The detailed information of active ingredients of ZYTLF.

**Additional file 2: Table S2. **List of ZYTLF-targets, PMOP-genes, and ZYTLF-PMOP overlapping genes.

**Additional file 3: Table S3.** Detail information of "Herb-Active ingredient-Overlapping gene" network.

**Additional file 4: Table S4. **Results of Gene Ontology (GO) terms and pathways.

**Additional file 5: Table S5.** The binding energies of ten core compounds and fifty key proteins.

## Data Availability

The datasets used and analyzed during the current study are available from the corresponding author on reasonable request.

## Abbreviations

PMOP: Postmenopausal osteoporosis
ZYTLF: Ziyin Tongluo Formula
TCMSP: Traditional Chinese medicine system pharmacology analysis platform
GO: Gene ontology
KEGG: Kyoto Encyclopedia of Genes and Genomes
BP: Biological processes
CC: Cellular components
MF: Molecular functions
OB: Oral bioavailability
DL: Drug-likeness
ADME: Absorption, distribution, metabolism, excretion
IL-6: Interleukin 6
TNF: Tumor necrosis factor
